# Different stages of Alzheimer’s disease with periodontitis: clinical features and potential mechanisms involving gingipains, neuropathological biomarkers and neurological damage

**DOI:** 10.3389/fnagi.2026.1737524

**Published:** 2026-04-20

**Authors:** Jing-hui Li, Teng-hong Lian, Peng Guo, Jing Li, Jing Qi, Ming-yue He, Dong-mei Luo, Ya-nan Zhang, Gai-fen Liu, Yue Huang, Wei-jia Zhang, Zi-jing Zheng, Hao Yue, Hui-ying Guan, Zhan Liu, Fan Zhang, Yao Meng, Wei Zhang

**Affiliations:** 1 Department of Neurology, Beijing Tiantan Hospital, Capital Medical University, Beijing, China; 2 Center for Cognitive Neurology, Department of Neurology, Beijing Tiantan Hospital, Capital Medical University, Beijing, China; 3 Department of Blood Transfusion, Beijing Tiantan Hospital, Capital Medical University, Beijing, China; 4 China National Clinical Research Center for Neurological Diseases, Beijing Tiantan Hospital, Capital Medical University, Beijing, China; 5 Department of Pharmacology, School of Medical Sciences, Faculty of Medicine & Health, UNSW Sydney, Sydney, NSW, Australia; 6 Center of Parkinson’s Disease, Beijing Institute for Brain Disorders, Beijing, China; 7 Beijing Key Laboratory on Parkinson Disease, Beijing, China

**Keywords:** Alzheimer’s disease, periodontitis, mild cognitive impairment, dementia, clinical features and potential mechanisms

## Abstract

**Background:**

Alzheimer’s disease (AD) and periodontitis are common in older adults aged over 65 years. However, the clinical features and mechanisms at the stages of mild cognitive impairment and dementia due to AD (AD-MCI and AD-D) remain unknown.

**Methods:**

In 110 patients with AD-MCI and AD-D, oral hygiene, periodontitis status, clinical symptoms and the levels of gingipain, neuropathological biomarkers and neurological damage indicators in cerebrospinal fluid were evaluated.

**Results:**

The frequency of periodontitis was 38.18% in the AD-MCI group and 67.27% in the AD-D group. The AD-MCI with periodontitis group had significantly worse cognition and lower β-amyloid (Aβ) level than those without periodontitis (all *p* < 0.05). The AD-D with periodontitis group had further impaired cognition and more severe neuropsychiatric symptoms. Gingipain K (K-GP) level was significantly correlated with Aβ, phosphorylated tau 199 and synaptosomal-associated protein 25 levels in the cerebrospinal fluid from AD-D with periodontitis group (all *p* < 0.05).

**Conclusion:**

Periodontitis is more prevalent and severe in AD patients. K-GP plays a significant role in AD with periodontitis, and is associated with AD pathology and impairs cognition at the MCI stage. K-GP contributes to the exacerbation of AD pathology and neurodegeneration, and is potentially involved in the aggravation of symptoms at the dementia stage.

## 1 Introduction

Alzheimer’s disease (AD) is the most common cognitive disorder among older adults aged over 65 years and characterized by symptoms include cognitive impairment, neuropsychiatric symptoms and compromised activities of daily living. Pathological biomarkers of AD include amyloid plaques and neurofibrillary tangles, which are composed of β-amyloid (Aβ) and phosphorylated tau (P-tau), respectively.

Periodontitis is the most common oral infectious disease with a prevalence of up to 50% in people over 65 years. It is a chronic inflammation of the periodontal supporting tissues caused by oral pathogens, and clinically characterized by gingival inflammation and bleeding, periodontal pocket formation and tooth mobility and loss.

Increasing evidence indicates that AD is associated with periodontitis (Aragón et al., 2018). AD patients exhibit worse periodontal clinical parameters, including probing depth, bleeding index and attachment loss than the cognitively normal population (Chen et al., 2017). Moreover, the more severe the periodontitis, the worse the cognitive function (Wang et al., 2019). Furthermore, AD patients present with more severe neuropsychiatric symptoms as periodontal attachment loss and dental plaque index worsen (Yang et al., 2021). However, most studies have focused on global cognitive and neuropsychiatric symptoms, and few studies have explored individual cognitive domains and neuropsychiatric symptoms in AD patients with periodontitis (AD-P). Additionally, no studies have analyzed AD-P across disease stages, including mild cognitive impairment (MCI) and dementia.

Gingipain (GP) is the most important virulence factor produced by *Porphyromonas gingivalis* (*Pg*), the most common pathogen in periodontitis (Sochocka et al., 2017). GP primarily consists of lysine-gingival protease (Gingipain K, K-GP) and arginine-gingival protease (Gingipain R, R-GP), which specifically recognize and hydrolyze lysine and arginine sites of host proteins, respectively. Both K-GP and R-GP are essential for *Pg* survival and play critical roles in evading host immune defenses and inducing pro-inflammatory factors. Animal experiments have indicated that mice exposed to GP exhibit significantly accumulated Aβ depositions in brain and impair cognition (Dominy et al., 2019; Ishida et al., 2017). Autopsy results have revealed a high degree of overlap between Aβ and GP in the brain regions of AD patients (Dominy et al., 2019; Ishida et al., 2017). Therefore, investigations on the relationship between GP and neuropathological biomarkers of AD have mainly relied on animal models and patient autopsies, and there is a lack of research on the relationship between GP and neuropathological biomarkers in the cerebrospinal fluid (CSF) of AD patients.

Synapses play a pivotal role in neuronal information transmission and memory. Synaptic loss is a core event at the early stage of AD and is closely related to the cognitive decline in patients (Rajendran and Paolicelli, 2018). Key synaptic proteins include synaptosomal-associated protein 25 (SNAP-25), synapsin and synaptophysin. SNAP-25, located at presynaptic membrane, mediates synaptic vesicle fusion, exocytosis and neurotransmitter release (Shin, 2014). SNAP-25 levels are elevated in CSF before symptoms occur in AD patients (Schindler et al., 2019), correlate with cognitive function and can predict disease progression in AD patients (Zhang et al., 2018). Synapsin is located at the membrane of synaptic vesicles, and is significantly decreased in AD mouse models. Synaptophysin, a presynaptic vesicle protein, is decreased by approximately 25% in patients with MCI due to AD (AD-MCI) and is associated with cognitive decline when reduced in hippocampus (Selkoe, 2002). Total tau (T-tau) is a biomarker of neurodegeneration, and its elevation in CSF indicates the increased neuronal loss. Therefore, both synaptic proteins and T-tau reflect neurological damage. However, no studies have investigated the relationship between GP and these neurological damage indicators in the CSF of AD patients.

In this study, we aimed to investigate the clinical features of AD patients with periodontitis at different disease stages and to elucidate the potential mechanisms involving gingipains, neuropathological biomarkers and neurological damage indicators.

## 2 Materials and methods

### 2.1 Ethics statement

This project was approved by the Review Board of Beijing Tiantan Hospital, Capital Medical University. All participants and their caregivers completed their written informed consent.

### 2.2 Participants

#### 2.2.1 Inclusion and exclusion criteria for AD patients

A total of 110 AD patients who met the criteria for AD-MCI or dementia due to AD (AD-D) according to the National Institute on Aging and Alzheimer’s Association criteria were recruited in this study (Albert et al., 2011; McKhann et al., 2011) (Supplementary Figure 1).

The exclusion criteria were as follows: (1) a history of stroke temporally associated with the onset or exacerbation of cognitive impairment, or the presence of multiple or extensive infarcts, or severe white matter hyperintensity. (2) the neurological diseases that might affect cognitive function besides AD, including frontotemporal degeneration, Lewy body disease, corticobasal degeneration, and Parkinson’s disease, etc. (3) the presence of non-neurological diseases or history of drug that might significantly affect cognitive function. (4) the articulation disorders and mental illnesses that might significantly affect emotional expression. (5) inflammatory and infectious diseases or recent antibiotic use. (6) the hearing loss, visual impairment or illiteracy that prevented the completion of all assessments in this study.

#### 2.2.2 Diagnostic criteria for periodontitis

Patients who met one of the following criteria were diagnosed with periodontitis: (1) two or more non-adjacent teeth had interproximal clinical attachment loss (CAL). (2) two or more non-adjacent teeth had buccal or lingual CAL ≥3 mm with periodontal pockets ≥3 mm (Papapanou et al., 2018; Tonetti et al., 2018).

In this study, patients were divided into the AD-P and the AD with no periodontitis (AD-nP) groups based on their periodontal status. According to the severity of AD, the AD-P group was subdivided into AD-MCI with periodontitis (AD-MCI-P) and AD-D with periodontitis (AD-D-P) groups, and the AD-nP group was subdivided into AD-MCI with no periodontitis (AD-MCI-nP) and AD-D with no periodontitis (AD-D-nP) groups.

### 2.3 Collection of demographic variables

Demographic variables, including age, gender, age of onset, disease duration, education level, body mass index, smoking and drinking were collected.

### 2.4 Assessments of clinical symptoms

#### 2.4.1 Overall cognitive function

Overall cognitive function was evaluated by the Mini-Mental State Examination (MMSE) (Folstein et al., 1975) and the Montreal Cognitive Assessment (MoCA) scales (Nasreddine et al., 2005). The lower the scores of the two scales, the worse the overall cognitive function.

#### 2.4.2 Cognitive domains

Multiple cognitive domains, including memory, visuospatial ability, attention, executive function and language, were assessed by a variety of rating scales, as detailed below.

Memory: Memory was evaluated by the Auditory Verbal Learning Test (AVLT) (Guo et al., 2009) and the Rey-Osterrieth Complex Figure (ROCF)-delayed recall (Shin et al., 2006). The lower scores of these scales indicate the poorer memory performance.

Visuospatial ability: Visuospatial ability was evaluated by the ROCF. A decreased score of this test implies a compromised visuospatial ability.

Attention: Attention was evaluated by the Symbol Digit Modalities Test (SDMT) (Fellows and Schmitter-Edgecombe, 2019), the Trail Making Test-A (TMT-A) (Wei et al., 2018), the Stroop Color-Word Test-A (SCWT-A) and the Stroop Color-Word Test-B (SCWT-B) (Bondi et al., 2002). The longer completion times or lower scores of these tests indicate the worse attention.

Executive function: Executive function was evaluated by the Trail Making Test-B (TMT-B) (Wei et al., 2018) and the Stroop Color-Word Test-C (SCWT-C) (Bondi et al., 2002). The decreased scores of these tests indicate the impaired executive function.

Language: Language was evaluated by the Verbal Fluency Test (VFT) (Mok et al., 2004) and the 30-item Boston Naming Test (BNT) (Katsumata et al., 2015). The lower scores of these scales indicate the more serious language impairment.

#### 2.4.3 Overall neuropsychiatric symptoms

Overall neuropsychiatric symptoms were evaluated using the Neuropsychiatric Inventory (NPI) (Cummings et al., 1994). The NPI-Caregiver Burden score reflects the level of distress experienced by caregivers due to the patient’s symptoms. The higher scores of the NPI indicate the more severe neuropsychiatric symptoms.

#### 2.4.4 Individual neuropsychiatric symptoms

Depressive and anxious symptoms were assessed by the 24-item Hamilton Depression Scale (HAMD) (Hamilton, 1960) and the 14-item Hamilton Anxiety Scale (HAMA) (Hamilton, 1959), respectively. Apathy and agitation were evaluated using the Modified Apathy Evaluation Scale (MAES) (Starkstein et al., 1992) and the Cohen-Mansfield Agitation Inventory (CMAI) (Koss et al., 1997), respectively. Overall sleep status was evaluated using the Pittsburgh Sleep Quality Index (PSQI) (Buysse et al., 1989). Excessive daytime sleepiness was valued by the Epworth Sleepiness Scale (ESS) (Johns, 1991). The higher scores of these scales indicate the more severe individual neuropsychiatric symptoms.

#### 2.4.5 Evaluations of oral hygiene and periodontitis variables

Oral hygiene was assessed using the Debris Index-Simplified (DI-S) and the Calculus Index-Simplified (CI-S). Periodontal variables, including probing depth (PD), bleeding index (BI), clinical attachment loss (CAL) and tooth loss, were evaluated by two experienced dental specialists. Prior to study initiation, the dental specialists completed standardized training and calibration sessions using a predefined examination protocol. During calibration, they jointly assessed a subset of participants to harmonize examination procedures and diagnostic criteria and to minimize inter-examiner variability. To minimize detection bias, the dental specialists were strictly blinded to participants’ cognitive status and diagnostic group allocation. Cognitive assessments and group assignment were performed by a separate team, and the dental specialists had no access to diagnostic information during periodontal assessment. All procedures and criteria were based on the fifth edition of the World Health Organization “Oral Health Surveys Basic Methods” (World Health Organization, 2013).

### 2.5 Collections and measurements of CSF samples

Drugs that improved cognitive function were withheld for 12–14 h if the patients’ conditions allowed. A total of 5 mL of CSF was obtained through lumbar puncture using strict aseptic techniques and collected into polypropylene tubes (Beijing JingkeHongda Biotechnology Co., Ltd., Beijing, China) between 7 and 10 a.m. under fasting conditions. CSF samples were centrifuged immediately (3000 rpm, 10 min) to remove cells and debris at 4 °C. Approximately 0.5 mL of supernatant from each CSF sample was aliquoted into separate Nunc cryotubes (Beijing JingkeHongda Biotechnology Co., Ltd., Beijing, China), and stored at −80 °C until analysis. Single-use aliquots were used for each measurement to avoid repeated freeze-thaw cycles and protein degradation.

The levels of K-GP and R-GP in the CSF from AD patients were measured by a double antibody sandwich method. The YS-S656 and YS-S742 kits were used for the measurements of K-GP and R-GP (Shanghai YSRIBIO Industrial Co., Ltd., Shanghai, China), respectively. The assays were performed strictly in accordance with the manufacturer’s instructions. The lower limit of detection was 3 pg/mL for K-GP and 3 pg/mL for R-GP, and all reported values were above these thresholds. The antibodies demonstrated no significant cross-reactivity with other relevant proteins. All CSF samples were analyzed in duplicate to ensure assay precision.

The measured neuropathological biomarkers of AD included Aβ42 and multiple forms of P-tau. The level of Aβ42 (CSB-E10684h kit, CUSABIO, Wuhan, China) and the levels of P-tau, including P-tau181 (Human Tau [pT181] phosphoELISA Kit, Invitrogen, Carlsbad, CA, USA), P-tau199 (Human Tau [pS199] ELISA Kit, Invitrogen, Carlsbad, CA, USA), P-tau231 (Human Tau [pT231] phosphoELISA Kit, Invitrogen, Carlsbad, CA, USA) and P-tau396 (Human Tau [pS396] ELISA Kit, Invitrogen, Carlsbad, CA, USA) were measured by enzyme-linked immunosorbent assay (ELISA).

The measured neurological damage indicators included synaptic proteins and T-tau. The levels of synaptic proteins, including SNAP-25 (Human SNAP-25 SimpleStep ELISA Kit ab256394, Abcam, Cambridge, UK), synapsin (Human Synapsin-1 ELISA Kit EK3879, Signalway Antibody LLC, College Park, MD, USA) and synaptophysin (Human Synaptophysin ELISA Kit NBP2-80283, Novus, Centennial, CO, USA) and T-tau (Human Tau Proteins ELISA Kit CSB-E12011h, CUSABIO, Wuhan, China) were measured by ELISA.

### 2.6 Data analyses

Statistical analyses were performed using SPSS Statistics 22.0 (IBM Corporation, New York, USA). A *p*-value < 0.05 was considered statistically significant.

Normally distributed continuous variables were expressed as means ± standard deviations and compared by two-tailed t-test. Non-normally distributed measurement data were presented as median (first quartile, third quartile) and were compared by non-parametric test. Chi-squared test was used to compare categorical variables. Bivariate correlation analysis, binary logistic regression and linear regression analysis were used to evaluate the relationships among the data measured. All models were adjusted for age, gender and years of education. Before building the regression models, all continuous independent variables were assessed for normality and homoscedasticity. Non-normally distributed variables were log-transformed to satisfy model assumptions. Multicollinearity was evaluated for all regression models, and no severe multicollinearity was detected.

## 3 Results

### 3.1 Frequency of periodontitis in AD patients

Among the 110 AD patients, 58 patients (52.72%) had periodontitis. In the AD-MCI group, 21 of 55 patients (38.18%) had periodontitis, whereas in the AD-D group, 37 of 55 patients (67.27%) had periodontitis.

### 3.2 Comparisons of demographic variables between the AD-nP and AD-P, and between the AD-MCI-P and AD-D-P groups

Demographic variables showed no significant differences between AD-nP and AD-P groups or between AD-MCI-P and AD-D-P groups (all *p* > 0.05) (Supplementary Table S1).

### 3.3 Comparisons of oral hygiene and periodontitis variables between the AD-MCI-P and AD-D-P groups

The AD-D-P group had significantly higher DI-S and CI-S scores and worse periodontitis variables, including probing depth, attachment loss and tooth loss than the AD-MCI-P group (all *p* < 0.05) (Table 1).

**Table 1:** Comparisons of oral hygiene and periodontitis variables between the AD-MCI-P and AD-D-P groups.

Oral hygiene and periodontitis variables	AD-MCI-P group (*n* = 21)	AD-D-P group (*n* = 37)	*p*
Probing depth (mm, x̄ ± SD)	1.92 ± 0.57	2.64 ± 1.05	0.006*
Clinical attachment loss [mm, median (Q1–Q3)]	2.13 (1.34, 3.30)	2.89 (2.39, 4.44)	0.007*
Bleeding index (mm, x̄ ± SD)	1.76 ± 0.72	1.81 ± 0.84	0.809
Tooth loss [number, median (Q1–Q3)]	2.00 (1.00, 3.00)	4.00 (1.00, 6.00)	0.02*
DI-S (score, x̄ ± SD)	14.48 ± 4.08	18.97 ± 6.69	0.007*
CI-S [score, median (Q1, Q3)]	13.00 (7.00, 18.50)	17.00 (11.00, 22.00)	0.018*

*p < 0.05. AD-MCI-P, mild cognitive impairment due to Alzheimer’s disease with periodontitis; AD-D-P, dementia due to Alzheimer’s disease with periodontitis; DI-S, debris index-simplified; CI-S, calculus index-simplified.

### 3.4 Clinical symptoms and their correlation with periodontitis variables in the AD-MCI-P and AD-D-P groups

**Figure 1:**
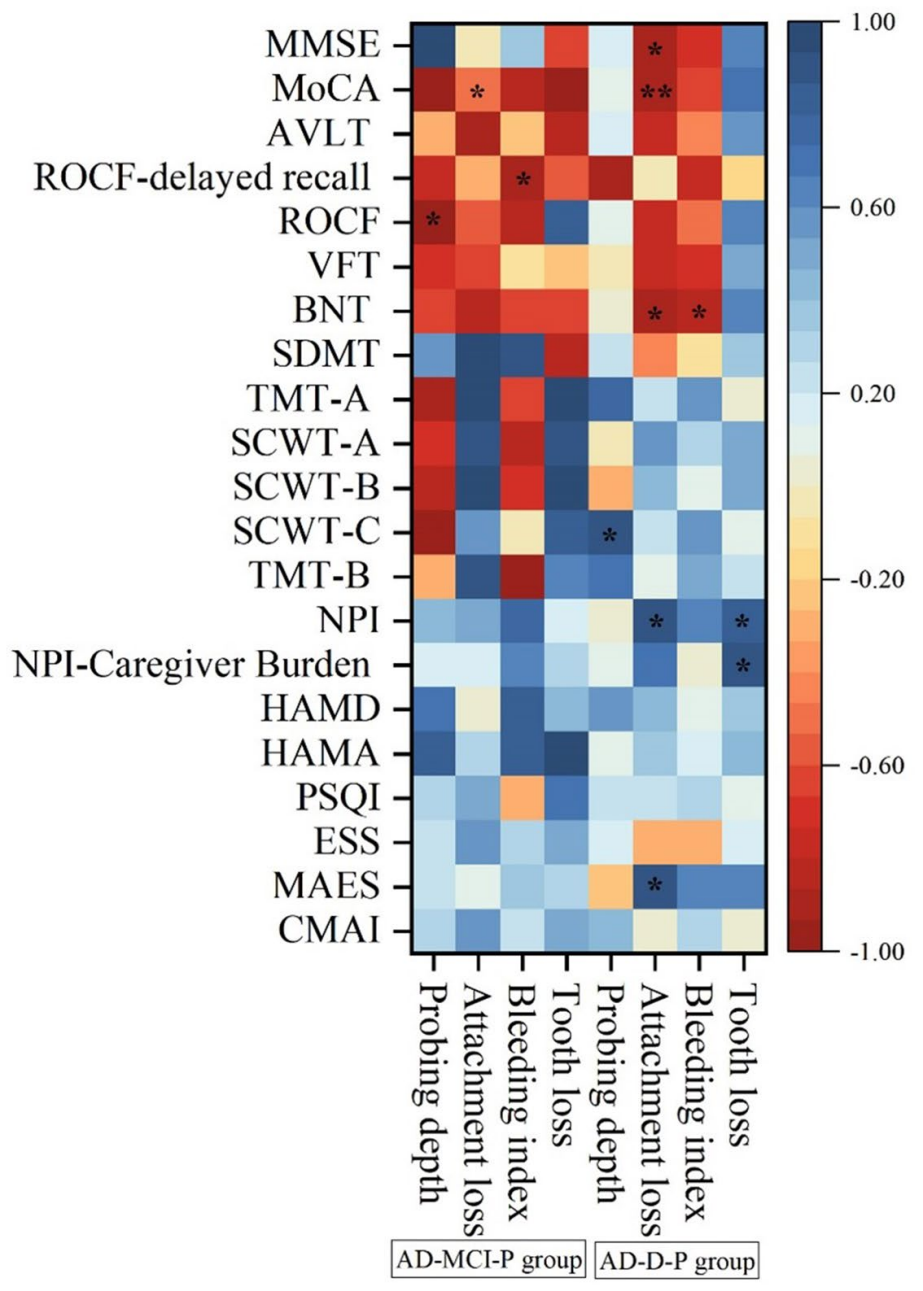
The correlations of periodontitis variables with cognitive function and neuropsychiatric symptoms in the AD-MCI-P and AD-D-P groups. *p < 0.05,**p < 0.01. MMSE, Mini-Mental State Examination; MoCA, montreal cognitive assessment; AVLT, auditory verbal learning test; ROCF, Rey-Osterrieth complex figure; VFT, verbal fluency test; BNT, Boston Naming Test; SDMT, symbol digit modalities test; TMT-A, Trail Making Test-A; SCWT-A, Stroop Color-Word Test-A; SCWT-B, Stroop Color-Word Test-B; SCWT-C, Stroop Color-Word Test-C; TMT-B, Trail Making Test-B; NPI, Neuropsychiatric Inventory; HAMD, Hamilton Depression Scale; HAMA, Hamilton Anxiety Scale; PSQI, Pittsburgh sleep quality index; ESS, Epworth Sleepiness Scale; MAES, Modified Apathy Evaluation Scale; CMAI, Cohen-Mansfield Agitation Inventory; AD-MCI-P, mild cognitive impairment due to Alzheimer’s disease with periodontitis; AD-D-P, dementia due to Alzheimer’s disease with periodontitis.

In the AD-MCI-P group, the scores of MMSE, MoCA and AVLT scales were significantly decreased compared with the AD-MCI-nP group (all *p* < 0.05) (Table 2). Furthermore, MoCA score had significantly negative correlation with attachment loss, ROCF score had significantly negative correlation with probing depth, and ROCF-delayed recall score had significantly negative correlation with bleeding index (all *p* < 0.05) (Figure 1). After adjusting for age, gender and education level, periodontitis was a significant predictor of the lower scores of the MoCA and ROCF-delayed recall scales (Supplementary Table S2).

**Table 2:** Comparisons of cognitive function between the AD-MCI-nP and AD-MCI-P groups, and between the AD-D-nP and AD-D-P groups.

Cognitive function	AD-MCI-nP group (*n* = 34)	AD-MCI-P group (*n* = 21)	*p*	AD-D-nP group (*n* = 18)	AD-D-nP group (*n* = 18)	*p*
Overall cognitive function						
MMSE (point, x̄ ± SD)	22.62 ± 3.64	18.43 ± 4.04	<0.01**	20.67 ± 3.63	12.68 ± 4.70	<0.001**
MoCA (point, x̄ ± SD)	17.91 ± 3.99	14.90 ± 4.43	0.012*	15.83 ± 5.13	9.14 ± 4.67	<0.001**
Cognitive domain						
Memory						
AVLT (point, x̄ ± SD)	15.44 ± 3.73	12.90 ± 4.30	0.025*	10.33 ± 3.03	6.81 ± 3.81	<0.001**
ROCF-delayed recall [point, median (Q1-Q3)]	11.50 (0.00, 23.00)	7.00 (0.50, 10.00)	0.103	0.00 (0.00, 7.00)	0.00 (0.00, 1.00)	0.766
Visuospatial ability						
ROCF [point, median (Q1–Q3)]	29.00 (21.50, 34.00)	30.00 (19.25, 33.50)	0.855	10.00 (5.37, 19.50)	8.00 (3.00, 13.50)	0.208
Attention						
SDMT (point, x̄ ± SD)	28.67 ± 12.86	31.88 ± 14.37	0.406	15.33 ± 13.44	11.92 ± 7.80	0.468
TMT-A [point, median (Q1–Q3)]	25.00 (24.00, 25.00)	25.00 (22.00, 25.00)	0.110	22.50 (21.00, 25.00)	18.00 (14.00, 25.00)	0.083
SCWT-A [point, median (Q1–Q3)]	50.00 (50.00, 50.00)	50.00 (50.00, 50.00)	0.255	50.00 (47.75, 50.00)	50.00 (46.50, 50.00)	0.261
SCWT-B [point, median (Q1–Q3)]	50.00 (49.75, 50.00)	50.00 (49.00, 50.00)	0.765	50.00 (45.00, 50.00)	48.00 (42.50, 50.00)	0.176
Executive function						
SCWT-C [point, median (Q1–Q3)]	48.50 (45.50, 50.00)	49.00 (46.50, 50.00)	0.894	39.00 (16.50, 47.25)	29.00 (0.00, 47.00)	0.315
TMT-B [point, median (Q1–Q3)]	25.00 (21.00, 25.00)	24.00 (18.00, 25.00)	0.374	15.50 (7.00, 21.25)	11.00 (5.50, 20.00)	0.189
Language						
VFT (point, x̄ ± SD)	38.24 ± 12.73	34.76 ± 8.03	0.269	21.22 ± 13.23	14.41 ± 6.86	0.014*
BNT (point, x̄ ± SD)	25.15 ± 3.31	24.00 ± 3.58	0.232	20.67 ± 4.56	17.49 ± 5.51	0.039*

*p < 0.05, **p < 0.01. AD-MCI-nP, mild cognitive impairment due to Alzheimer’s disease with no periodontitis; AD-MCI-P, mild cognitive impairment due to Alzheimer’s disease with periodontitis; AD-D-nP, dementia due to Alzheimer’s disease with no periodontitis; AD-D-P, dementia due to Alzheimer’s disease with periodontitis; MMSE, Mini-Mental State Examination; MoCA, Montreal Cognitive Assessment; AVLT, Auditory Verbal learning Test; ROCF, Rey-Osterrieth Complex Figure; SDMT, Symbol Digit Modalities Test; TMT-A, Trail Making Test-A; SCWT-A, Stroop Color-Word Test-A; SCWT-B, Stroop Color-Word Test-B; SCWT-C, Stroop Color-Word Test-C; TMT-B, Trail Making Test-B; VFT, Verbal Fluency Test; BNT, Boston Naming Test.

In the AD-D-P group, the scores of MoCA, AVLT, VFT and BNT scales were significantly decreased compared with the AD-D-nP group (all *p* < 0.05) (Table 2). Moreover, the scores of MMSE and MoCA scales were significantly and negatively correlated with attachment loss, and BNT score was significantly and negatively correlated with attachment loss and bleeding index (all *p* < 0.05) (Figure 1). In the AD-D-P group, the scores of NPI and MAES scales were significantly elevated compared with the AD-D-nP group (all *p* < 0.05) (Table 3). Furthermore, the scores of NPI and MAES scales were significantly and positively correlated with attachment loss, and the scores of NPI and NPI-Caregiver Burden scales were significantly and positively correlated with tooth loss (all *p* < 0.05) (Figure 1). After adjusting for age, gender and education level, periodontitis predicted the lower scores of MMSE, MoCA and BNT scales, and higher scores of NPI and MAES scales (Supplementary Table S3).

**Table 3:** Comparisons of neuropsychiatric symptoms between the AD-MCI-nP and AD-MCI-P groups, and between the AD-D-nP and AD-D-P groups.

Neuropsychiatric symptoms	AD-MCI-nP group (*n* = 34)	AD-MCI-P group (*n* = 21)	*p*	AD-D-nP group (*n* = 18)	AD-D-P group (*n* = 37)	*p*
Overall neuropsychiatric symptoms						
NPI [point, median (Q1–Q3)]	1.00 (0.00, 7.25)	2.00 (0.00. 6.00)	0.915	3.50 (1.00, 6.00)	9.00 (1.50, 11.00)	0.012*
NPI- Caregiver Burden [point, median (Q1-Q3)]	0.00 (0.00, 1.00)	0.00 (0.00, 1.00)	0.238	1.00 (0.75, 2.00)	1.00 (0.00, 1.00)	0.232
Individual neuropsychiatric symptoms						
HAMD (point, x̄ ± SD)	4.88 ± 3.76	5.00 ± 4.96	0.921	7.33 ± 7.06	4.78 ± 4.69	0.116
HAMA (point, x̄ ± SD)	4.26 ± 2.84	5.10 ± 4.48	0.403	4.72 ± 3.68	5.41 ± 4.70	0.591
PSQI (point, x̄ ± SD)	5.65 ± 5.40	6.95 ± 5.05	0.377	4.83 ± 3.07	4.78 ± 3.47	0.959
ESS [point, median (Q1, Q3)]	3.00 (1.00, 5.00)	4.00 (2.00, 7.00)	0.107	1.50 (1.00, 5.00)	2.00 (1.00, 5.50)	0.413
MAES [point, median (Q1, Q3)]	4.50 (2.00, 7.00)	5.00 (1.00, 7.50)	0.814	3.00 (2.00, 5.00)	14.00 (8.00, 19.00)	<0.001**
CMAI [point, median (Q1–Q3)]	29.00 (29.00, 30.00)	30.00 (29.00, 31.50)	0.155	29.00 (29.00, 35.25)	30.00 (29.00, 35.25)	0.734

*p < 0.05, **p < 0.01. AD-MCI-nP, mild cognitive impairment due to Alzheimer’s disease with no periodontitis; AD-MCI-P, mild cognitive impairment due to Alzheimer’s disease with periodontitis; AD-D-nP, dementia due to Alzheimer’s disease with no periodontitis; AD-D-P, dementia due to Alzheimer’s disease with periodontiti; NPI, Neuropsychiatric Inventory; HAMD, Hamilton Depression Scale; HAMA, Hamilton Anxiety Scale; PSQI, Pittsburgh sleep quality index; ESS, Epworth Sleepiness Scale; MAES, Modified Apathy Evaluation Scale; CMAI, Cohen-Mansfield Agitation Inventory.

### 3.5 Comparisons of CSF levels of GP, neuropathological biomarkers and neurological damage indicators between the AD-nP and AD-P groups

Compared with the AD-nP group, the AD-P group displayed significantly decreased Aβ42 level and increased K-GP, SNAP-25 and T-tau levels in CSF (all *p* < 0.05) (Table 4).

**Table 4:** Comparisons of the levels of GP, neuropathological biomarkers and neurological damage indicators in CSF between the AD-nP and AD-P groups, and between the AD-MCI-P and AD-D-P groups.

	AD-nP group (*n* = 52)	AD-P group (*n* = 58)	*p*	AD-MCI-P group (*n* = 21)	AD-D-P group (*n* = 37)	*p*
GP						
K-GP (pg/ml, x̄ ± SD)	94.57 ± 25.82	110.49 ± 31.83	<0.005**	101.09 ± 15.42	115.84 ± 37.29	0.090
R-GP (pg/ml, x̄ ± SD)	181.69 ± 60.73	199.97 ± 45.88	0.076	154.69 ± 64.45	174.04 ± 41.01	0.167
Neuropathological biomarkers						
Aβ42 [ng/ml, median (Q1, Q3)]	0.59 (0.44, 0.91)	0.44 (0.30, 0.90)	0.020*	1.03 (0.34, 1.69)	0.37 (0.26,0.64)	0.005**
P-tau181 [pg/ml, median (Q1, Q3)]	64.49 (30.06, 85.52)	68.91 (51.66, 87.43)	0.212	53.35 (36.25,81.42)	69.72 (60.04, 87.80)	0.026*
P-tau199 [pg/ml, median (Q1, Q3)]	8.15 (5.29, 10.89)	9.17 (4.55, 11.81)	0.157	5.72 (3.01, 9.12)	10.99 (4.82, 12.36)	0.010*
P-tau231 (pg/ml, x̄ ± SD)	52.27 ± 30.32	59.89 ± 41.91	0.282	51.94 ± 31.21	53.85 ± 36.15	0.840
P-tau396 [pg/ml, median (Q1, Q3)]	54.22 (31.50, 70.71)	55.99 (27.31, 106.41)	0.075	51.82 (30.48, 109.91)	60.17 (26.31, 100.55)	0.806
Neurological damage indicators						
SNAP-25 [pg/ml, median (Q1, Q3)]	55.21 (36.29, 83.87)	95.26 (62.17, 138.83)	<0.001**	72.01 (53.58, 96.98)	128.69 (75.36, 146.56)	0.007*
Synapsin (ng/ml, x̄ ± SD)	0.15 ± 0.09	0.13 ± 0.084	0.323	0.13 ± 0.08	0.13 ± 0.09	0.907
Synaptophysin (pg/ml, x̄ ± SD)	405.03 ± 297.97	526.59 ± 407.31	0.080	472.87 ± 392.90	557.08 ± 417.47	0.454
T-tau (pg/ml) [pg/ml, median (Q1, Q3)]	72.10 (51.73, 120.20)	94.80 (75.29, 122.60)	0.041*	81.69 (17.20, 99.23)	88.92 (65.43, 111.03)	0.105

*p < 0.05, **p < 0.01. GP, gingipain; CSF, cerebrospinal fluid; AD-nP, Alzheimer’s disease with no periodontitis; AD-P, Alzheimer’s disease with periodontitis; AD-MCI-P, mild cognitive impairment due to Alzheimer’s disease with periodontitis; AD-D-P, dementia due to Alzheimer’s disease with periodontitis. K-GP, gingipain K; R-GP, gingipain R; Aβ, β amyloid; P-tau, phosphorylated tau; SNAP-25, Synaptosomal-associated protein 25; T-tau, total tau.

### 3.6 The relationships between CSF levels of GP and neuropathological biomarkers in the AD-MCI-P and AD-D-P groups

Compared with the AD-MCI-P group, the AD-D-P group had significantly decreased Aβ42 level and increased P-tau181 and P-tau199 levels in CSF (all *p* < 0.05) (Table 4).

**Figure 2:**
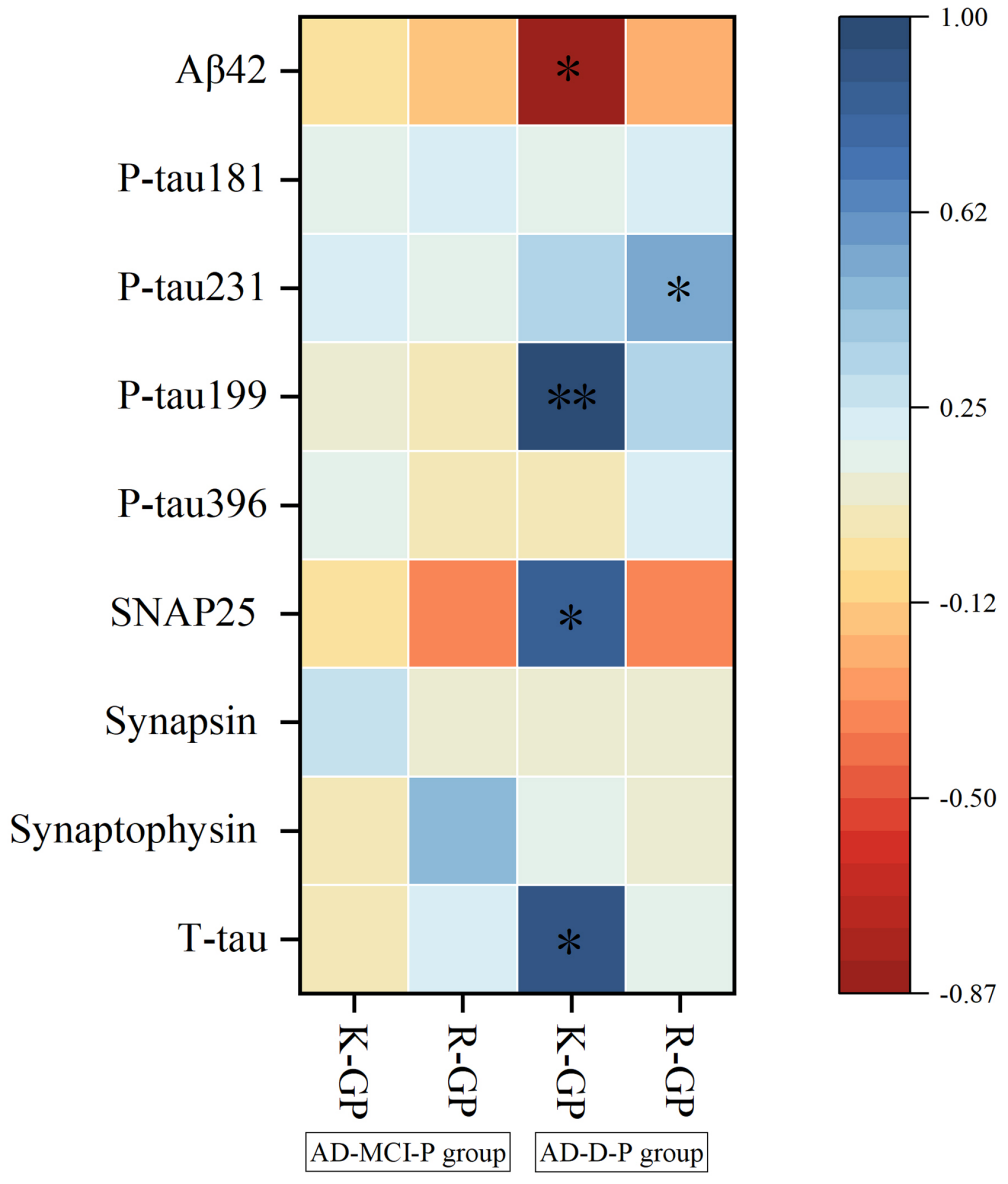
The correlation analyses between the CSF levels of GP and neuropathological biomarkers, and between the CSF levels of GP and neurological damage indicators in AD-MCI-P and AD-D-P groups. *p < 0.05, **p<0.01. Aβ, β amyloid; P-tau, phosphorylated tau; SNAP-25, Synaptosomal-associated protein 25; T-tau, total tau; K-GP, gingipain K; R-GP, gingipain R; AD-MCI-P, mild cognitive impairment due to Alzheimer’s disease with periodontitis; AD-D-P, dementia due to Alzheimer’s disease with periodontitis.

Although the AD-MCI-P group showed no significant correlation between the levels of GP and neuropathological biomarkers in CSF, the AD-D-P group presented that K-GP level was significantly and negatively correlated with Aβ42 level, and positively correlated with P-tau199 level in CSF (all *p* < 0.05) (Figure 2). Meanwhile, R-GP level was significantly and positively correlated with P-tau231 level in CSF (*p* < 0.05) (Figure 2).

In AD-D patients, after adjusting for potential confounding factors of age, gender, education level and disease duration, elevated K-GP level was still significantly associated with the decreased Aβ42 level and increased P-tau199 level in CSF (Supplementary Table S4).

### 3.7 The relationship between CSF levels of GP and neurological damage indicators in the AD-MCI-P and AD-D-P groups

Compared with the AD-MCI-P group, the AD-D-P group had **s**ignificantly elevated SNAP-25 level in CSF (*p* < 0.05) (Table 4).

Although the AD-MCI-P group showed no significant correlation between the levels of GP and neurological damage indicators in CSF, the AD-D-P group displayed that K-GP level had significantly positive correlations with SNAP-25 and T-tau levels in CSF (all *p* < 0.05) (Figure 2). After adjusting for confounding factors of age, gender, education level and disease duration, the increased K-GP level was significantly associated with the increased SNAP-25 levels in CSF in the AD-P group (Supplementary Table S4).

## 4 Discussion

Recent studies have shown that periodontitis is closely associated with AD, but the prevalence of periodontitis in AD patients has not been well documented. In this study, we found a high prevalence of periodontitis among AD patients (52.72%), with a significantly higher rate at the AD-D stage (67.27%) compared to the AD-MCI stage (38.18%). These findings indicate that periodontitis is highly prevalent in AD and its prevalence increases with disease progression from MCI to dementia.

Furthermore, we observed no significant differences in demographic variables between the AD-P and AD-nP groups or between the AD-MCI-P and AD-D-P groups (Supplementary Table S1). This suggests that demographic factors did not confound the following results and establishes a basis for comparability between groups.

In this study, the AD-D group had worse oral hygiene, which might explain the greater severity of periodontitis in this group. Moreover, probing depth, clinical attachment loss and tooth loss were more severe in the AD-D-P group than in the AD-MCI-P group (Table 1). Overall, oral hygiene was poorer, and probing depth and clinical attachment loss were greater in AD patients than those in the cognitively normal population (Martande et al., 2014). However, oral hygiene and periodontal profiles in AD patients with or without periodontitis at different disease stages have seldom been described. A limitation of this study is the lack of information on periodontitis staging and grading, as well as the data on disease duration. Staging and grading can more precisely reflect disease severity and rate of progression, while duration information is crucial for understanding cumulative exposure to chronic inflammation. The absence of these data may limit our ability to evaluate the potential dose-dependent effects of periodontitis.

Periodontal parameters, such as probing depth and clinical attachment loss, reflect the severity and progression of periodontitis and represent long-term accumulated damage to periodontal tissues. Tooth loss increases the likelihood of cognitive decline (Fang et al., 2018) and dementia, and it has been linked to the shrinkage of the brain regions responsible for memory, learning and overall cognition (Kobayashi et al., 2018). The observed increase in tooth loss in this study may reflect participants’ long-term history of periodontitis. However, its sensitivity as an indicator of current inflammatory activity is limited. In our study, patients in the AD-D group had worse oral hygiene and more severe periodontitis, likely due to their compromised ability to manage oral hygiene independently and effectively.

Currently, no studies have investigated the effects of periodontitis on individual cognitive domains in AD patients at different disease stages. In this study, the AD-MCI-P group showed significant impairment in overall cognitive function and in the memory domain compared to the AD-MCI-nP group (Table 2). Additionally, aggravated periodontitis was correlated with poorer memory and overall cognition after adjusting for gender, age and education level (Supplementary Table S2). Episodic memory is the first cognitive domain impaired in typical AD and remains the most frequently compromised one in patients who develop dementia. GP led to a significant reduction in hippocampal neurons (Leira et al., 2019), thus providing a potential basis for periodontitis-related memory impairment. Therefore, at the MCI stage of AD, periodontitis might cause damage to memory-related brain regions, thereby contributing to overall cognitive decline. As AD progressed to the dementia stage, periodontitis appeared to further impair other cognitive domains, such as language (Table 2), and was significantly associated with poorer overall cognition in the AD-D-P group after adjusting for confounding factors (Supplementary Table S3). Language dysfunction is an important symptom of AD. Naming impairment worsened with AD progression and was correlated with the atrophy of anterior temporal lobe (Corbett et al., 2012), which was adjacent to hippocampus. We hypothesize that as AD advances to the dementia stage, GP may spread from hippocampus to anterior temporal lobe, thereby impairing language function.

No prior studies have examined the effects of periodontitis on the individual neuropsychiatric symptoms across different stages of AD. In this study, no differences in neuropsychiatric symptoms were found between the AD-MCI-nP and AD-MCI-P groups (Table 3), nor were any significant correlations observed between periodontitis variables and neuropsychiatric symptoms in the AD-MCI-P group (Figure 1). This suggests that periodontitis does not play a significant role in neuropsychiatric symptoms at the MCI stage. We propose that this may be because the AD-MCI group exhibited a relatively shorter disease duration and milder periodontitis compared to the AD-D group (Supplementary Table S1), thus limiting the impact of periodontitis on neuropsychiatric symptoms at this stage. However, the AD-D-P group exhibited significantly worse overall neuropsychiatric symptoms, particularly more severe apathy (Table 3). Furthermore, the aggravation of periodontitis was significantly associated with the deterioration of overall neuropsychiatric symptoms and apathy in the AD-D-P group, independent of age, gender and education level (Supplementary Table S3). Apathy, as the most frequent neuropsychiatric symptom at the moderate and severe stages of AD, has been found to be associated with increased Aβ deposition in frontal lobe and anterior cingulate gyrus (Stella et al., 2014). Therefore, periodontitis may promote Aβ deposition in these brain regions, thereby contributing to the prominent apathy and more severe neuropsychiatric symptoms at the dementia stage of AD.

However, it should be noted that although this study found that the current periodontal status in AD patients with periodontitis was associated with poorer clinical symptoms, the cross-sectional design cannot determine whether this association reflects recent inflammatory activity or the cumulative effects of long-term chronic exposure. Notably, a prospective cohort study (Karaduran et al., 2023) demonstrated that the presence of periodontitis was associated with an accelerated rate of cognitive decline over a 6-month follow-up period. This finding suggests that severe periodontal inflammation may not only correlate with neurodegeneration but also potentially exacerbate disease progression, aligning with the clinical parameters observed in our cohort.

While our findings, supported by longitudinal evidence, suggest that periodontitis may accelerate cognitive decline, the potential bidirectional relationship (Zhang et al., 2026) cannot be ignored. The progressive cognitive impairment and loss of manual dexterity associated with AD often lead to neglected oral hygiene and diminished self-care capacity. This decline in oral health maintenance promotes plaque accumulation and exacerbates periodontal inflammation, potentially creating a vicious cycle in which neurodegeneration and periodontitis mutually reinforce each other. In addition, individuals with cognitive impairment may be less likely to receive regular and professional dental care, or may have difficulty completing comprehensive periodontal treatment and long-term maintenance follow-up. Future research should adopt longitudinal designs with repeated measurements of both oral health and cognitive function beginning at the earlier stages of AD to clarify the temporal sequence of these associations.

A previous *in vitro* study showed that *Pg* invaded neurons and secreted GP, resulting in neuronal damage associated with AD (Haditsch et al., 2020). In this study, the K-GP level in CSF was significantly elevated in the AD-P group compared to the AD-nP group (Table 4), suggesting that K-GP produced and released by *Pg* entered the brains of AD patients. Moreover, as AD progressed to the dementia stage, the K-GP level in CSF did not show a significant increase, although an upward trend was observed. In contrast, the R-GP level in CSF did not differ significantly between the AD-P and AD-nP groups or between the AD-MCI-P and AD-D-P groups (Table 4). A previous *in vivo* study demonstrated that an orally administered K-GP inhibitor was more effective than an R-GP inhibitor in clearing *Pg* from brain and protecting hippocampal neurons (Dominy et al., 2019). Hence, K-GP may play a more prominent role than R-GP in mediating the influence of periodontitis on AD.

We next explored the relationship between periodontitis and AD neuropathological biomarkers in CSF. In the cognitively normal elderly population with periodontitis, greater clinical attachment loss was associated with increased Aβ deposition in brain (Kamer et al., 2015). Moreover, GP increased P-tau generation by promoting neuroinflammation and aggravated neuronal damage, thereby accelerating cognitive decline (Tobore, 2019). In this study, the Aβ42 level in CSF was significantly decreased in the AD-P group compared to the AD-nP group (Table 4). Furthermore, the AD-D-P group showed a significantly decreased Aβ42 level along with significantly elevated levels of P-tau181 and P-tau199 in CSF (Table 4). Decreased CSF Aβ42 level is generally considered to reflect increased Aβ deposition in brain. As the core biomarker of AD, it has been found that P-tau181 in CSF predicted the cognitive decline in patients at preclinical, MCI and dementia stages, and was associated with the severity of AD (Vos et al., 2013). P-tau199 in CSF was significantly elevated and correlated with the degree of neurodegeneration (Suárez-Calvet et al., 2020). Therefore, our findings suggest that periodontitis is associated with the increased Aβ deposition in the brains of AD patients. Furthermore, across the disease spectrum from MCI to dementia, periodontitis was correlated with the elevated Aβ deposition and P-tau level, potentially reflecting accelerated neurodegeneration and cognitive deterioration. This is supported by our observation that in the AD-D-P group, the increased K-GP level in CSF was associated with the enhanced Aβ deposition and P-tau199 production (Supplementary Table S4), indicating that K-GP may be a key virulence factor promoting the formation of amyloid plaques and neurofibrillary tangles at the dementia stage of AD. It is important to note that our findings regarding GP are based on observational data. These results should be interpreted as hypothesis-generating and require further validation through longitudinal and mechanistic studies.

In this study, the levels of SNAP-25 and T-tau in CSF were significantly elevated in the AD-P group compared with the AD-nP group (Table 4). Additionally, the SNAP-25 in CSF level was significantly elevated in the AD-D-P group compared with the AD-D-nP group (Table 4). After adjusting for confounding factors, K-GP was significantly associated with aggravated synaptic damage at the dementia stage of AD (Supplementary Table S4). Synaptic dysfunction emerges at the early stages of AD, and the alteration of SNAP-25 in CSF reflects this synaptic damage; elevated CSF SNAP-25 level was associated with cognitive decline in AD patients (Öhrfelt et al., 2016). Similarly, T-tau is a well-established biomarker of neurodegeneration that indicates AD progression and increases as MCI progresses to dementia. Consequently, our results demonstrate that the AD-D-P group experiences more severe synaptic damage and neurodegeneration. This suggests that at the dementia stage, periodontitis may be linked to more severe neurological damage and advanced AD progression, a pattern that differs from the observations at the MCI stage.

K-GP-dependent heme acquisition is essential for the virulence of *Pg* (Smalley et al., 2007), and thus K-GP is considered a greater contributor to virulence than R-GP (O’Brien-Simpson et al., 2001). Studies have indicated that K-GP may trigger the production of numerous pro-inflammatory factors in brain, including tumor necrosis factor-*α*, interleukin (IL)-1β, IL-6 and C-reactive protein (Dominy et al., 2019). Hence, we hypothesize that at the MCI stage of AD, K-GP may contribute to Aβ deposition, potentially via microglial activation (Memedovski et al., 2020), thereby possibly facilitating the release of inflammatory factors and contributing to cognitive impairment. Previous study indicated that K-GP disrupted blood–brain barrier by compromising the integrity of vascular endothelial junctions (Nonaka et al., 2022). Consequently, we speculate that although the K-GP level in CSF was not significantly elevated (Table 4), its persistence in brain may lead to chronic blood–brain barrier disruption. This could facilitate further K-GP infiltration, creating a vicious cycle that exacerbates the accumulations of Aβ42 and P-tau199 and amplifies synaptic damage, thereby aggravating both cognitive and neuropsychiatric symptoms in AD patients. However, these specific cellular and molecular cascades remain to be elucidated through future longitudinal studies and experimental models.

Due to the absence of a cognitively normal control group or a non-AD MCI group, the observed associations between periodontitis and AD pathology/clinical symptoms may be contextually limited to the AD disease spectrum. Specifically, the relationship between periodontitis and core AD pathologies (Aβ and Tau) as well as clinical symptoms may be influenced by pre-existing neuroinflammation and blood–brain barrier dysfunction inherent to AD. Consequently, our findings primarily suggest how periodontitis may act as an aggravating factor within the context of established AD pathology.

Although the total sample size was sufficient for the primary analyses, the number of participants in the subgroups was relatively small. This may result in limited statistical power for detecting the weaker associations between periodontitis and AD and performing more complex interaction analyses. Consequently, our findings regarding subgroup differences and specific biomarker correlations should be considered exploratory and require validation in future larger cohorts.

The absence of a cognitively normal control group or a non-AD MCI group is a limitation of this study, which restricts the generalizability of our findings. Future studies need to incorporate age-matched healthy controls to validate the specificity of these results. Furthermore, due to the cross-sectional design of this study, causal conclusions cannot be drawn. The findings reported here require validation through longitudinal cohorts and experimental studies. Moreover, this study has the relatively small number of CSF samples. Collecting CSF from AD patients was particularly challenging because they were often elderly and presented with the conditions of low intracranial pressure, spinal deformity or bone hyperplasia. In future, we plan to expand the collection of CSF samples to include not only AD patients but also appropriate control groups, such as individuals with normal cognitive function and those with subjective cognitive decline.

## 5 Conclusion

The prevalence of periodontitis is 38.18% at the MCI stage and 67.27% at the dementia stage of AD. AD-P patients at the dementia stage have worse oral hygiene and more severe periodontitis than those at the MCI stage. Periodontitis is associated with poorer memory and overall cognition at the MCI stage, and correlated with deficits in a broader range of cognitive domains as well as more severe neuropsychiatric symptoms at the dementia stage of AD. K-GP exerts a more dominant role in AD with periodontitis than R-GP. The elevated K-GP level in CSF is observed alongside the increased Aβ deposition at the MCI stage and associated with greater accumulation of both Aβ and P-tau199, as well as SNAP-25 at the dementia of AD. These associations suggest a potential link to the worsening of cognitive and neuropsychiatric symptoms.

Neurologists and dentists should take joint action to regularly evaluate oral hygiene and periodontitis for AD patients, assess the functions of overall cognition and cognitive domains at the MCI stage, and attach importance to both cognitive and neuropsychiatric symptoms at the dementia stage. Interventions targeting periodontitis may offer additional benefits in delaying disease progression, but their definitive causal effect requires validation through future prospective interventional studies. For patients with severe dementia, clinicians should educate caregivers on managing daily oral hygiene. The novel findings from this study are helpful for understanding clinical features and potential mechanisms involving GP, neuropathological proteins and neurological damage biomarkers at different stages of AD with periodontitis and provide potential therapeutic targets for drug development.

## Data Availability

The raw data supporting the conclusions of this article will be made available by the authors, without undue reservation.
