# Supplementary material for: Different stages of Alzheimer’s disease with periodontitis: clinical features and potential mechanisms involving gingipains, neuropathological biomarkers and neurological damage
Source: Front Aging Neurosci. 2026 Apr 20;18:1737524. doi: 10.3389/fnagi.2026.1737524 (PMC13136118; doi:10.3389/fnagi.2026.1737524)
Supplement: Supplementary file 1 [file Data_Sheet_1.docx]

**Supplementary Table S1 Comparisons of demographic variables**

**between AD-nP and AD-P groups, and between AD-MCI-P and AD-D-P groups**

|  | **AD-nP group**  **(n=52)** | **AD-P group (n=58)** | **P** | **AD-MCI-P group**  **(n=21)** | **AD-D-P group （n=37）** | **P** | |
| --- | --- | --- | --- | --- | --- | --- | --- |
| Age (years,‾x ± SD) | 61.98 ± 10.94 | 63.29 ± 10.03 | 0.513 | 62.81 ± 10.08 | 63.57 ± 10.13 | 0.785 | |
| Male (n, %) | 19(36.54%) | 21(36.21%） | 0.971 | 8(38.09%) | 13(35.14%) | 0.822 | |
| Age of onset [years, median (Q1, Q3)] | 59.50(53.25, 66.75) | 59.00(51.00, 66.25) | 0.594 | 59.00(53.00, 65.50) | 59.00(49.25, 67.50) | 0.728 | |
| Disease duration [months, median (Q1, Q3)] | 24.00(13.50, 36.00) | 36.00(24.00, 48.00) | 0.064 | 24.00(21.00, 48.00) | 37.50(24.00, 60.00) | 0.133 | |
| Education level [years, median (Q1, Q3)] | 12.00(9.00, 15.00) | 12.00(9.00, 15.00) | 0.944 | 12.00(7.50, 15.00) | 12.00(9.00, 15.00) | 0.980 |  |
| Body mass index (‾x ± SD) | 23.71 ± 2.11 | 22.99 ± 2.50 | 0.112 | 24.09 ± 3.62 | 22.56 ± 2.76 | 0.079 |  |
| Smoking (n, %) | 10(19.23%) | 16(27.59%) | 0.303 | 1(4.76%) | 3(8.12%) | 0.889 |  |
| Drinking (n, %) | 10(19.23%) | 12(20.69%) | 0.849 | 6(28.57%) | 6(16.22%) | 0.264 |  |

Abbreviations: AD-nP, Alzheimer’s disease with no periodontitis; AD-P, Alzheimer’s disease with periodontitis; AD-MCI-P, mild cognitive impairment due to Alzheimer’s disease with periodontitis; AD-D-P, dementia due to Alzheimer’s disease with periodontitis.

**Supplementary Table S2 Associations between cognitive function and periodontitis in AD-MCI patients**

|  | **OR (95% CI)** | **p** |
| --- | --- | --- |
| MoCA | 0.852(0.728, 0.997) | 0.046* |
| ROCF-delayed recall | 0.928(0.862, 0.999) | 0.046* |
| ROCF | 1.037(0.979, 1.098) | 0.213 |

*: P<0.05. The model was adjusted for age, gender and education level.

Abbreviations: AD-MCI, mild cognitive impairment due to Alzheimer’s disease;

AD-D-P, dementia due to Alzheimer’s disease with periodontitis; MoCA, Montreal

Cognitive Assessment; ROCF, Rey-Osterrieth Complex Figure.

**Supplementary Table S3 Associations between cognitive function/neuropsychiatric symptoms and periodontitis in AD-D patients**

|  | **OR (95% CI)** | **p** |
| --- | --- | --- |
| MMSE | 0.455(0.248, 0.836) | 0.011* |
| MoCA | 0.712(0.577, 0.879) | 0.002** |
| BNT | 0.862(0.758, 0.980) | 0.023* |
| SCWT-C | 0.976(0.946, 1.008) | 0.139 |
| NPI | 1.223(1.035, 1.446) | 0.018* |
| MAES | 1.523(1.146, 2.024) | 0.004** |

*: P<0.05, **: P<0.01. The model was adjusted for age, gender and education level.

Abbreviations: AD-D, dementia due to Alzheimer’s disease.

MMSE, Mini-Mental State Examination; MoCA, Montreal Cognitive Assessment;

BNT, Boston Naming Test; SCWT-C: Stroop Color-Word Test-C; NPI,

Neuropsychiatric Inventory; MAES, Modified Apathy Evaluation Scale.

**Supplementary Table S4 Associations between the levels of neuropathological biomarkers/neurological damage indicators**

**and K-GP in CSF in AD-D patients**

|  | **β (95% CI)** | **p** |
| --- | --- | --- |
| Aβ42 | -29.238(-43.866, -14.609) | <0.001** |
| P-tau 199 | 5.011 (1.555, 8.468) | 0.005* |
| SNAP25 | 0.265 (0.012, 0.519) | 0.040* |
| T-tau | -0.088 (-0.296, 0.119) | 0.395 |

*: P<0.05, **: P<0.01. The model was adjusted for age, gender, education level and disease duration.

Abbreviations: AD-D, dementia due to Alzheimer’s disease; K-GP, gingipain K; Aβ, β amyloid; P-tau,

phosphorylated tau; SNAP-25, Synaptosomal-associated protein 25; T-tau, total tau.

**
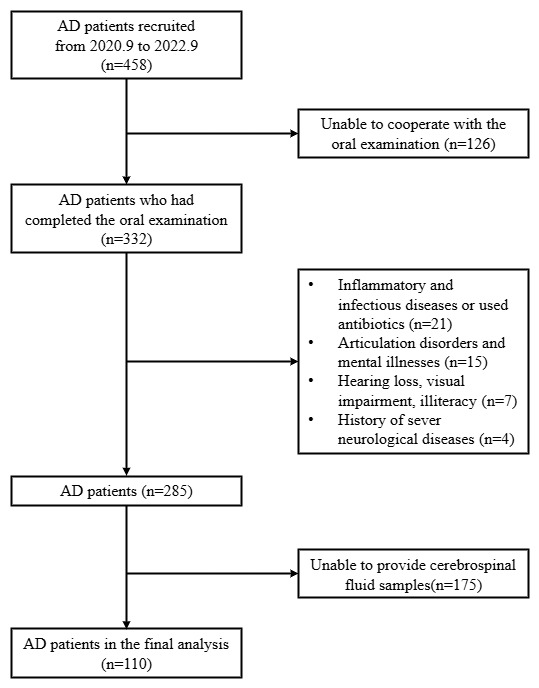
**

**Supplementary Figure 1** **Study flow chart.** Abbreviations: AD, Alzheimer’s disease.
